# 恶性复杂中央气道病变的气管镜介入治疗

**DOI:** 10.3779/j.issn.1009-3419.2016.12.08

**Published:** 2016-12-20

**Authors:** 洪武 王, 楠 张, 冬妹 李, 梅梅 陶, 洁莉 张, 珩 邹, 云芝 周, 素娟 梁, 秀云 白

**Affiliations:** 100028 北京，煤炭总医院肿瘤科 Department of Medical Oncology, Meitan General Hospital, Beijing 100028, China

**Keywords:** 中央型肺癌, 冷冻, 氩等离子体凝固, 内支架, 气管镜介入治疗, Central cell carcinoma, Cryosurgery, Argon plasma coagulation, Stent, Interventional bronchoscopies

## Abstract

**背景与目的:**

复杂气道狭窄是临床处理的难题，尤其是恶性气道肿瘤。本研究旨在总结112例恶性复杂气道狭窄气管镜介入治疗的效果，以探讨热消融、冷冻等不同方法在患者中的应用价值。

**方法:**

回顾性分析112例恶性中央型气道病变患者，均累及隆突附近的气管下段、双侧支气管开口至少两个部位的病变，年龄22岁-90岁，其中鳞癌(squamous carcinoma, SQ)55例、腺癌(adenocarcinoma, AD)16例、腺样囊性癌(adenocystic carcinoma, ACC)15例、转移性癌(metastasis tumor, MT)10例、小细胞肺癌(small cell lung carcinoma, SCLC)及混合癌(mixted carcinoma, MC)各8例，均在硬质气管镜或电子气管镜下进行氩等离子体凝固(argon plasma coagulation, APC)、电圈套器、二氧化碳(CO2)冷冻等气管镜下介入治疗。

**结果:**

112例恶性气道病变患者进行了460例次气管镜治疗，各组均以冷冻和APC最常用，MT中还有内支架置入。球囊导管扩张主要用于ACC和AD，圈套器和粒子植入常用于SCLC。术前气管狭窄以MT组最重，同一癌症中SQ及ACC支气管的阻塞程度均重于气管。术前MC、MT和SCLC三组卡氏体能状态评分(Karnofsky performance score, KPS)明显降低，而气促评分却明显升高。经多种方法综合治疗后，除MC外，其他五组气管和支气管狭窄均有明显改善。除AD组外，其他五组KPS均明显升高、气促评分均明显下降。术后随访，患者总的生存时间是15个月，中位生存时间10个月。其中以ACC(28.4个月)和AD(21.7个月)的中位生存时间最长，而以SCLC(8.9个月)和MC(7.4个月)最短。

**结论:**

针对累及多个部位的恶性中央型气道病变，应采取以冷冻和APC为主的联合治疗方法，无论对气管还是支气管，均有良好的治疗作用，快速、安全，值得推广。

## Abstract (en)

**Background and objective:**

It is very difficult to deal with the comprehensive central airway diseases, especially the malignant airway obstruction. The aim of this study is to explore the clinical effects of multiple interventional bronchoscopies for the treatment of 112 cases with comprehensive malignant central airway diseases so as to evaluate the clinical applications of thermoablation and cryosurgery.

**Methods:**

The 112 cases with malignant airway diseases retrospectively reviewed for bronchoscopic interventions performed in more than two lesions of carina and/or bifurcation. The age was from 22 to 90 years, which including 55 cases with squamous carcinoma (SQ), 16 adenocarcinoma (AD), 15 adenocystic carcinoma (ACC), 10 metastasis tumor (MT), 8 small cell lung carcinoma (SCLC) and 8 mixed carcinoma (MC). All bronchoscopic interventions such as argon plasma coagulation (APC), cryosurgery, electronic snare and stenting were performed under rigid bronchoscopy combined with electric bronchoscopy in general anesthesia.

**Results:**

The 460 bronchoscopic procedures were successfully performed in 112 cases. Cryosurgery and APC were the most used in every group. Stent was more common in patients with MT, balloon dilation was more common in patients with ACC and AD. Electric snare and radioactive seeds implantation were frequently used in cases with SCLC. Trachea stricture is more severe in MT than that in others, while bronchus stricture is more severe than that of trachea in SQ and ACC group. Karnofsky performance score (KPS) is lower and shortness of breath score (SBS) is higher in MC, MT and SCLC group. Trachea and bronchus stricture is improved after comprehensive treatment in 5 groups except of mixed group. KPS increased and SS decreased significantly in 5 groups except of adenocarcinoma. In follow-up, the overall survival time (OS) is 15 months and median survival time is 10 months, especially OS is 28.4 months in ACC and 21.7 months in AD, 8.9 months in SCLC and 7.4 months in mixed group.

**Conclusions:**

APC combination with Cryosurgery and other bronchoscopic interventions are indicated for the treatment of complex or difficult airway diseases whether they are located in trachea or bronchus. It is a safe and rapid during procedure.

中央型肺癌是临床最常见的肺癌类型。如果病变侵及气管下段、隆突及双侧主支气管开口等多个部位，往往失去手术治疗时机，放疗、化疗短期内也难奏效，常危及生命，易引起窒息，临床治疗非常困难。硬质镜下介入治疗是解决此类气道病变最快速的方法之一^[1]^，但操作过程中也有极大的风险。近年来我们治疗了逾千例恶性气道肿瘤，其中有112例较为复杂、危重，但经过综合治疗，均取得理想效果，在此与同行们共享临床经验。

## 1 临床资料

### 1.1 患者资料

回顾性分析自2006年8月-2013年8月在我院接受治疗的112例中央型气道病变，均累及隆突附近的气管下段、双侧支气管开口至少两个部位，年龄22岁-90岁，其中鳞癌(squamous carcinoma, SQ)55例、腺癌(adenocarcinoma, AD)16例、腺样囊性癌(adenocystic carcinoma, ACC)15例、转移性癌(metastasis tumor, MT)10例、小细胞肺癌(small cell lung carcinoma, SCLC)及混合癌(mixted carcinoma, MC)各8例，均在硬质气管镜或电子气管镜下进行氩等离子体凝固(argon plasma coagulation, APC)、电圈套器、CO2冷冻、内支架置入等气管镜下介入治疗，如表 1所示。本组112例气道疾病患者共进行了460例次气管镜治疗(1个病例可能需多次治疗)，以冷冻和APC最常用，MT中还有内支架置入。球囊导管扩张主要用于ACC和AD，圈套器和粒子植入常用于SCLC。

**1:** 460例次气管镜治疗分析(例次) Analyses in 460 cases with interventional bronchoscopies

Pathology	*n*	CO_2_ Cryo (%)	APC (%)	Stents (%)	Balloon dilatation (%)	Snare (%)	^125^I implantation (%)	Drug injection (%)
SQ	203	114 (56.2)	160 (78.8)	13 (6.4)	0 (0)	25 (12.3)	15 (7.4)	26 (12.8)
ACC	83	33 (39.7)	34 (41.0)	5 (6.0)	7 (8.4)	10 (12.0)	7 (8.4)	7 (8.4)
AD	56	15 (26.8)	35 (62.5)	11 (19.6)	5 (8.9)	7 (12.5)	2 (3.6)	4 (7.1)
MT	52	18 (34.6)	24 (46.1)	23 (44.2)	0 (0)	5 (9.6)	0 (0)	3 (5.8)
MC	41	28 (68.3)	31 (75.6)	1 (2.4)	0 (0)	2 (4.9)	1 (2.4)	2 (4.9)
SCLC	25	14 (56.0)	15 (60.0)	3 (12.0)	0 (0)	6 (24.0)	3 (12.0)	2 (8.0)
SQ: squamous carcinoma; AD: adenocarcinoma; ACC: adenocystic carcinoma; MT: metastasis tumor; SCLC: small cell lung carcinoma; MC: mixed carcinoma.								

### 1.2 操作方法

所用硬质气管镜为德国Tutlingen产品Karl Storz。所用电子气管镜为日本PENTAX公司产品EPM 3500。硬质气管镜术前做胸部计算机断层扫描(computed tomography, CT)(必要时行增强CT)及肺功能，由内镜医师及麻醉师进行评估。术中需监测血氧饱和度、心电图、血压及呼吸运动等。

患者平卧手术床上，肩背部底下放一垫子，以使头后仰，便于硬质镜插入。充分氧合好后给予患者全身静脉麻醉。

经口插入硬质气管镜，通过硬质气管镜的侧孔连接麻醉机或高频喷射通气，维持足够的氧饱和度。同时，在不停呼吸机的情况下通过硬质气管镜后孔进行各种操作。术中通过电子气管镜活检孔插入APC电极、冷冻探头、电圈套器等设备进行介入治疗。

对病情较轻的病例或非首次治疗则单纯在电子气管镜下进行治疗，所有患者均采用监控下全身静脉麻醉，毋须用呼吸机机械通气。操作按常规进行。

气管镜介入治疗：APC所用设备为德国产CESEL 3000型。将APC探针通过电子气管镜活检孔伸出气管镜插入端(能见到探针标志为准)，在距病灶0.5 cm以内时开始烧灼。APC输出功率为30 W-50 W，氩气流量为0.8 L/min-1.6 L/min。烧灼过程中勿需停止吸氧，但吸氧浓度需低于40%，持续时间不能太长，并不断用活检钳取出碳化凝固的组织(碳化的组织易燃着火)。

冷冻机采用北京库兰医疗设备有限公司生产的冷冻治疗仪K300型和德国ERBE。电圈套器型号为南京微创公司生产。被膜金属支架为江苏西格玛公司生产。软式可弯曲冷冻探头直径1.9 mm-2.3 mm，探针末端长度5 mm。冷源为液态二氧化碳。将冰冻探头的金属头部放在肿瘤表面或推进到肿瘤内，冷冻5 s-10 s，使其周围产生最大体积的冰球，在冷冻状态下将探头及其粘附的肿瘤组织取出，必要时再插入探头，直至将腔内的肿瘤全部取出。冻取后如有出血，则结合APC止血。若将冰冻探头的金属头部放在病灶表面持续冷冻1 min-3 min，称为冻融。

电圈套器型号为南京微创公司生产。将电圈套器连接在高频电刀上。通过电子气管镜的活检通道将电圈套器套扎在肿瘤上，然后启动高频电凝，将肿瘤切下。再用光学活检钳或冷冻将切下的肿瘤取出。

放射性粒子植入：在气管镜引导下，通过WANG氏穿刺针将131I粒子植入到气道周围的淋巴结或肿块内，根据治疗计划系统决定植入粒子数量，一般为5个-15个粒子。

其他如球囊导管扩张、粘膜下药物注射等根据操作常规进行。

### 1.3 统计学分析

采用SPSS 22.0统计软件进行数据处理，计量资料组间差异比较使用*t*检验。*P* < 0.05为差异有统计学意义。

## 2 结果

由表 2可见，术前气管的狭窄程度MT分别明显重于SQ、ACC和AD(*P* < 0.01)，而与MC和SCLC无明显差异(*P* > 0.05)，其他各组间亦无明显差异(*P* > 0.05)。但同一种癌症中SQ及ACC支气管的阻塞程度均重于气管(*P* < 0.01)，而其他四组均无明显差异(*P* > 0.05)。术前MC、MT和SCLC三组KPS分别明显低于SQ、ACC和AD三组(*P* < 0.01)，而气促评分(shortness of breath score, SBS)却明显升高(均*P* < 0.01)。经多种方法综合治疗后，除MC气道狭窄无明显下降外，其他癌组气管和支气管狭窄均有明显改善。除AD组KPS治疗后无明显升高外，其他各组KPS均明显升高、气促评分均明显下降。

**2:** 气管镜治疗前后的病情变化 Changes of patient's condition after bronchoscopic therapy

Pathology	Trachea stricture (%)				Bronchus stricture (%)				KPS (%)				Shortness of breath score		
	*n*	Before	After		*n*	Before	After		*n*	Before	After		*n*	Before	After
SQ	96	46.1±2.6^*^	20.1±1.4		234	57.8±1.7^*^	31.2±1.5		175	60.5±1.7^*^	70.8±1.5		175	2.6±0.1^*^	1.5±0.1
ACC	55	47.7±3.4^*^	26.9±2.4		76	61.7±3.0^*^	40.4±2.6		76	73.6±1.5^*^	78.5±1.2		76	2.3±0.1^*^	1.6±0.1
AD	28	43.6±5.3^*^	19.3±2.6		50	53.9±4.7^*^	37.1±4.2		52	72.1±2.2	77.9±2.3		52	2.1±0.1^*^	1.3±0.1
MT	13	63.1±6.4^*^	25.7±5.3		45	64.8±3.7^*^	22.2±3.5		23	53.9±2.9^*^	73.0±3.0		23	3.4±0.2^*^	1.7±0.2
MC	11	48.6±9.2	28.2±7.6		45	73.1±3.5^*^	32.3±4.4		25	57.6±3.5^*^^*^	68.0±2.6		25	3.0±0.2^*^	2.0±0.2
SCLC	13	56.1±8.2^*^	21.5±3.6		39	68.6±6.2^*^	37.0±4.4		20	47.5±3.5^*^	60.0±2.6		20	3.4±0.1^*^	2.2±0.2
There are multiple lesions in a same patient. Bronchi are including bilateral main bronchus and right intermedius bronchus. ^*^P < 0.01, ^*^^*^P < 0.05, compared between before and after treatment in a same group; KPS: Karnofsky performance score.															

治疗后经6个月-3年的随访，6组患者的生存期有明显差异。患者总的生存时间是15个月，中位生存时间10个月。其中以ACC(28.4个月)和AD(21.7个月)的中位生存时间最长，而以MC(7.4个月)和SCLC(8.9个月)最短。

## 3 讨论

累及隆突附近的复杂性中央型气道恶性肿瘤，常危及生命，手术难以切除，放/化疗难以短时间内缓解症状。近年来由于硬质气管镜的应用，特别是气管镜介入技术的发展，可以快速清除气道内的恶性肿瘤，畅通气道，减轻梗阻症状，明显改善患者预后^[2, 3]^。硬质气管镜插入管不但有侧孔与呼吸机相连，还有粗大的介入通道允许软性气管镜及其他器械进入气道内，大大拓宽了其应用范围，可在直视下进行支架释放、激光消融、氩等APC和冷冻等操作^[4]^。

我们的研究结果显示，六组癌症患者术前气管的狭窄程度均约50%左右，MT(为63.1%)分别明显重于SQ、ACC和AD(*P* < 0.01)，而与MC和SCLC无明显差异(*P* > 0.05)，其他各组间亦无明显差异(*P* > 0.05)。各组支气管的阻塞约为60%左右，同一癌症中SQ(57.8%)及ACC(61.7%)均重于气管(*P* < 0.01)，而其他四组均无明显差异(*P* > 0.05)。术前MC、MT和SCLC病情较重，三组KPS分别明显低于SQ、ACC和AD三组(*P* < 0.01)，而SBS却较高(均*P* < 0.01)。经多种方法综合治疗后，除MT组外，其他五组气管和支气管狭窄均有明显改善，绝大多数病变狭窄程度均可减轻50%以上。除AD组外，其他五组KPS均明显升高、SBS均明显下降。AD病变大多位于支气管^[5]^，因此肿瘤消除后，患者的症状可能短期内没有得到明显改善。

要快速缓解气道内的梗阻症状，就要准确判断气道内病变的性质。根据病变部位，可分为管内型、管壁型、管外型和混合型。对管内型、管壁型主要采取以圈套、冻取和热消融治疗为主的治疗方法，可快速消除瘤负荷，改善症状。而对管外型可采取内支架置入、放射性离子植入为主的治疗方法。

本组112例气道恶性肿瘤患者共进行了460例次气管镜治疗，以冷冻和APC最常用。转移癌多为混合型，先消融治疗腔内肿瘤，若还有气道狭窄，可再置入内支架。ACC和腺癌也多为混合型，且病变段较长，可先用球囊导管扩张，再辅以内支架等治疗。SCLC、ACC和鳞癌多为管内型或有蒂的肿瘤，可先用电圈套器套扎组织或肿瘤，若切除的瘤体较大，在软镜下难以取出，且易脱落引起窒息，则在硬镜下结合钳取、冻取等，取出较为简单，不会引起窒息。对基底较宽或肿瘤表面血管丰富或已有出血的肿瘤，则先用APC止血，然后冻切或硬质镜铲除，再随时结合APC止血。比单用软镜操作更为简单、快捷。

硬镜铲除是利用半弧形的硬镜前端直接将肿瘤铲下，再利用活检钳将肿瘤取出，这是软镜所不具备的功能。术中先用哪种方法，或需结合应用哪些方法，需根据病变部位和类型而定。一般应先打通气管，可在硬镜下利用大号活检钳咬取、高频电刀圈套器套取、CO_2_冻取及APC烧灼等方式快速疏通气道。对同时侵犯双侧支气管的病变，应先打通阻塞程度轻的一侧，然后再处理阻塞程度重的一侧。治疗过程中应不断将气道下段的积血吸出，以免堵塞下段支气管口。一般对气道狭窄75%以上的恶性肿瘤以硬质镜治疗为佳，治疗后气管阻塞程度、气促指数和KPS评分均有明显改善^[2]^。

放射性粒子植入常用于对放疗敏感的SCLC，也可用于管壁上有肿块或管外有淋巴结转移的病变^[6]^。

作者早期Y型支架需在C型臂引导下放置，而现在大多在硬质镜下放置则更为安全、准确，患者无痛苦。

操作过程中需密切注意各种并发症的发生。如冻取过程中需注意大出血^[7]^、热消融过程中需注意吸氧浓度及气道内着火等^[8]^，内支架置入后需注意分泌物潴留及肉芽肿形成等。

既往认为难以纠正的低氧血症是硬质镜检查的禁忌证，也是麻醉医师不愿给予全麻的重要原因之一。实际不然，静脉全麻后快速插入硬质镜，并连接机械通气，可快速纠正缺氧，稳定病情，避免窒息等严重并发症的发生^[9]^。所以，对病情危重、病变复杂的气道肿瘤，最好在全麻硬镜下进行。近年来作者应用软镜引导下插入硬质镜的方法(王氏插入法)，可同时给氧，并在1 min内插入硬镜，减少了软、硬镜间的切换，操作极为快捷方便，为抢救患者赢得了宝贵的时间。

(感谢河北联合大学流行病学教研室徐应军教授为本组数据做了统计学分析)
